# Giant central nervous system tuberculoma in pediatric patients: surgical case series

**DOI:** 10.1007/s00381-021-05091-1

**Published:** 2021-03-06

**Authors:** Xiao Xiao, Qiang Li, Yan Ju

**Affiliations:** Department of Neurosurgery, West China Hospital of Sichuan University, No. 37 Guoxue Road, Chengdu, 610041 Sichuan Province People’s Republic of China

**Keywords:** Tuberculoma, Hydrocephalus, Meningitis

## Abstract

Central nervous system tuberculoma is rare and challenging situation. Clinical records of patients with pathologically proven tuberculoma were retrospectively reviewed. Clinical presentation, lesion location, radiological characteristics, perioperative and surgical management, and outcome is summarized and analyzed. Eight patients were included and there was one girl. Age ranged from 3 to 14 years with mean age 9.8 years. Clinical duration ranged from 20 days to 2 years, and 3 patients had previous lung tuberculosis with anti-TB treatment. The lesion was in cerebellum in 6 cases, including 1 involving basal ganglia and 1 involving thalamus. The lesion was in basal ganglia, thalamus, and third ventricle in 1 case, and in T12-L1 spinal cord in another. Cerebellar lesion was resected via paramedian suboccipital approach in 5 patients, basal ganglia lesion via trans-cortical frontal horn approach in 2 patients, and intra-spinal lesion via trans-laminar approach in 1 patient. Follow-up ranged from 10 to 24 months. Of the 8 patients, 6 returned to normal life. One patient had cerebellar lesion resected and the thalamic lesion reduced in size after anti-TB treatment. One patient died from TB spreading. Our data showed that most patients can be successfully treated by resection of the lesion. Low T2 signal, ring shaped enhancement and peripheral edema strongly suggest tuberculoma. Empirical anti-TB treatment should be initiated perioperatively.

## Introduction

Tuberculosis (TB) is a common chronic infectious disease in developing countries and is a major threat to public health. According to WHO’s data in 2011. There were 47,698 new tuberculosis case notifications, including 1378 in China. In 2012, there were around totally 530,000 TB cases in children and 74,000 deaths among them [1]. TB is usually caused by *Mycobacterium tuberculosis* and manifests as lung TB, meningitis, tuberculoma, lymphadenopathy, or gastrointestinal TB.

The involvement of central nervous system (CNS-TB) is the most severe form of tubercle bacillus infection. CNS-TB accounts for about 5–10% of all patients, with a mortality up to 20% depending on the clinical stage [2, 3] Most CNS-TB patients are treated medically. Surgical treatment is possible only when the space occupying the tuberculoma or abscess is apparent, or when critical hydrocephalus is pending. However, CNS tuberculoma is a rare entity in the pediatric population, and very few cases have been reported [4–6]. Giant tuberculoma is even more rare. In this article, we review and report eight pediatric patients with giant tuberculomas involving the CNS. The clinical features and management of the giant tuberculomas are discussed.

## Methods

In pediatric neurosurgical department, the clinical records of patients with pathologically proven tuberculoma were retrospectively reviewed. The duration was from January 2017 to December 2018. Our definition of “giant” tuberculoma included the following: (1) intracranial lesion more than 3 cm in maximum diameter; or (2) intraspinal lesion sized more than half of the cross section of the spinal cord.

Clinical presentation, lesion location, radiological characteristics, perioperative and surgical management, and outcomes were summarized and analyzed. This study was reviewed and approved by the Ethnic Committee of West China Hospital. All patients’ guardians provided informed consent for the publication of these cases.

After the careful evaluation of the patient’s history and undergoing a physical examination, each patient underwent computed tomography (CT) scan for head and thorax and magnetic resonance imaging (MRI) for the CNS. For those with severe hydrocephalus, emergent extraventricular drainage (EVD) via frontal horn was performed under general anesthesia. Dehydration, corticosteroids, fluid therapy, and the use of prophylactic anti-epileptic drug were conducted. Standard anti-TB therapy with HRZE regimen (isoniazid, rifampicin, pyrazinamide, and ethambutol) was resumed or initiated, if TB was previously diagnosed or seriously suspected.

Each patient underwent microsurgical resection of the most prominent symptom-producing or space-occupying lesion under general anesthesia. Postoperative computed tomography was performed to evaluate the surgical field and hydrocephalus. Ventriculoperitoneal shunt (VPS) was considered if the hydrocephalus deteriorated after clamping of the extraventricular drainage.

## Results

Eight patients were included (Table 1). Only one girl was included. The patients were in the age range of 3–14 years old with a mean age of 9.8 years old. Four patients were of Yi ethnicity from the remote rural areas of Sichuan Province. Two were of Tibetan ethnicity from Tibet. Two were of Han Ethnicity from local rural places. The lesion was in the cerebellum in six cases, including one involving the basal ganglia and one involving the thalamus. The lesion was in the basal ganglia, thalamus, and third ventricle in one case, and in the T12-L1 spinal cord in another.

**Table 1:** Clinical features of our patients

No. and gender	Age (years)	Ethnicity	Presentation	Duration	Location	Imaging characteristic	Preop TB	Hydrocephalus	Treatment	Postop course	FU and prognosis
1.M	6	Yi	Fever headache vomiting	4 months	Cerebellum	Ring enhancement, solitary, low T2, edema	No	Yes	Resection	Good	Good
2.M	6	Yi	Headache vomiting	3 months	Cerebellum	Ring enhancement, multiple, low T2, edema	Yes lung	Yes	Resection	Good	Good
3.M	13	Yi	Headache vomiting	2 months	Cerebellum	Ring enhancement, multiple, low T2, edema	Yes lung	Yes	Resection	Good	Good
4.M	10	Tibetan	Headache	2 years	Cerebellum	Ring enhancement, multiple, low T2, edema	Unknown	Yes EVD	Resection	VPS, 2nd EVD dissemination	Dismal
5.M	3	Tibetan	Headache hemiparalysis	7 months	Cerebellum, thalamus	Ring enhancement, multiple, low T2, edema	No	Yes	Resection (cerebellum)	Fair	Fair
6.F	14	Yi	Headache hemiparalysis	3 months	Basal ganglia, cerebellum	uneven enhancement, multiple, mid-low T2, edema	Yes lung	No	Resection (basal ganglia)	Good	Good
7.M	14	Han	Headache fever hemiparalysis	20 days	Basal ganglia, third ventricle	Ring enhancement, multiple, low T2, edema	No	Yes	Resection (basal ganglia)	Good	Good
8.M	13	Han	Lower limb pain paralysis	2 months	T12-L1	Ring enhancement, solitary, low T2, edema	No	No	Resection	Good	Good

*EVD* external ventricular drainage, *VPS* ventriculoperitoneal shunt, *TB* tuberculosis, *FU* follow-up

Clinical duration ranged from 20 days to 2 years, and only three patients had previous lung TB with anti-TB treatment. All patients except for the one with intraspinal lesion presented with headache. Among the patients, three had concomitant nausea and vomiting; two had low fever and sweating; and three had hemiparesis associated with basal ganglia or thalamic involvement. The patient with T12-L1 lesion presented with bilateral lower limb pain and decreased strength.

**Fig. 1:**
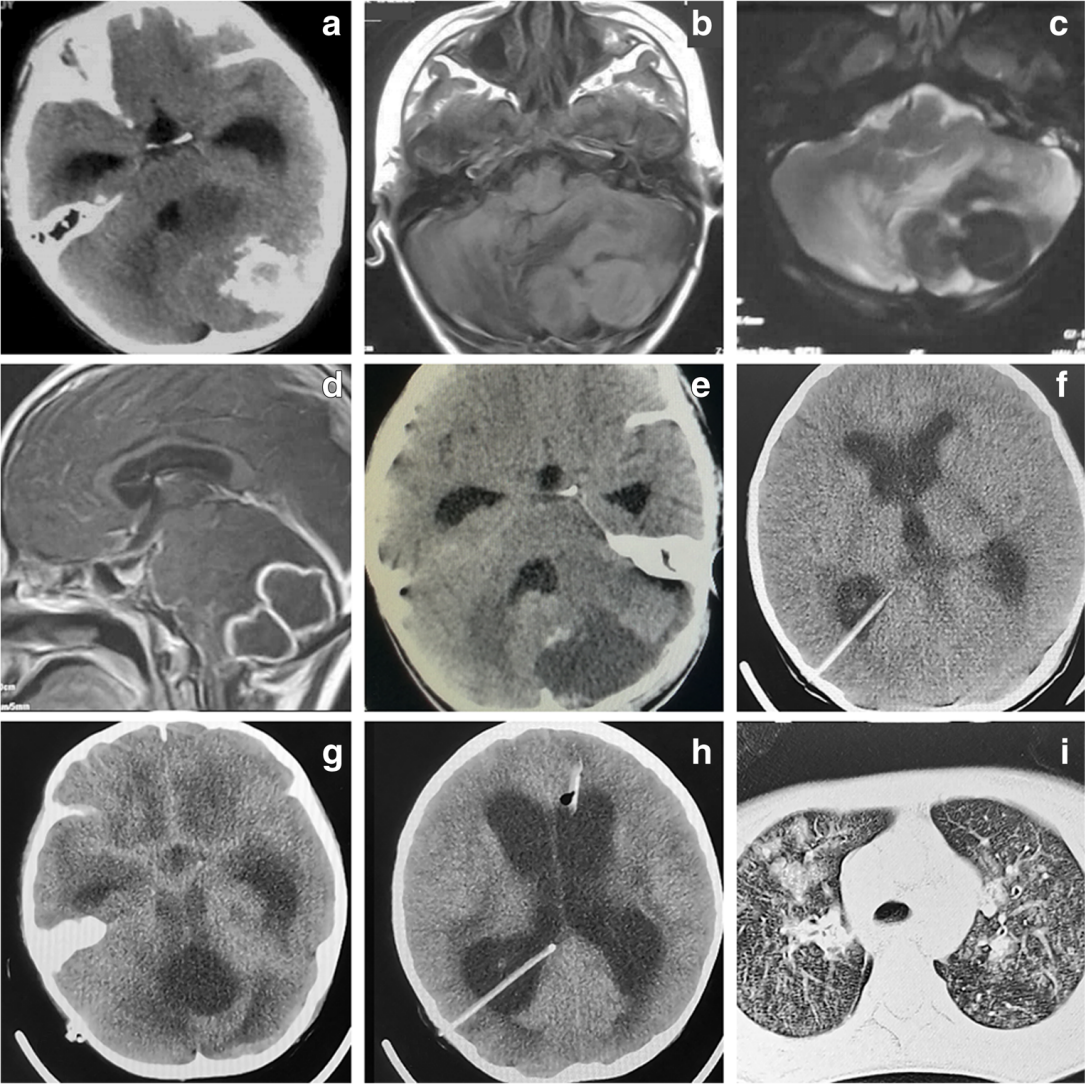
Images of Patient 4 with preoperative EVD. **a**, CT scan showing big calcified lesion in left cerebellum and hydrocephalus. **b**, MR T1 showing the lesion is iso-intense and lobulated. **c**, MR T2 showing low intensity, typical of caseous necrosis and marked peripheral edema. **d**, MR T1 after gadolinium showing ring shaped enhancement of the lesion and prepontine cistern enhancement, suggestive of meningitis. **e**, CT scan after lesion resection showing complete removal of the tuberculoma. **f**, CT scan showing the ventricles shrunk and sulci re-appeared after VPS. **g-h**, end-stage CT scan showing accumulation of hyperdense pus in major cisterns and still enlarged ventricles after 2nd EVD. **i**, chest CT scan after deterioration showing lung infection suspected of spreading TB

Preoperative CT scan showed calcification in most tuberculomas. MR imaging showed that six patients had multiple lesions, and two had solitary lesion. All giant tuberculomas appeared as low intensity in T2-weighted imaging with prominent perilesional edema, and ring enhancement appeared in seven patients after gadolinium injection (Fig. 1). Only one patient with basal ganglia mass showed uneven enhancement. Six patients had hydrocephalus, but only one needed emergent EVD. The one with EVD was rated as Grade III according to Modified Vellore grading system, and all the other six patients barely met the criteria of Grade I [7].

**Fig. 2:**
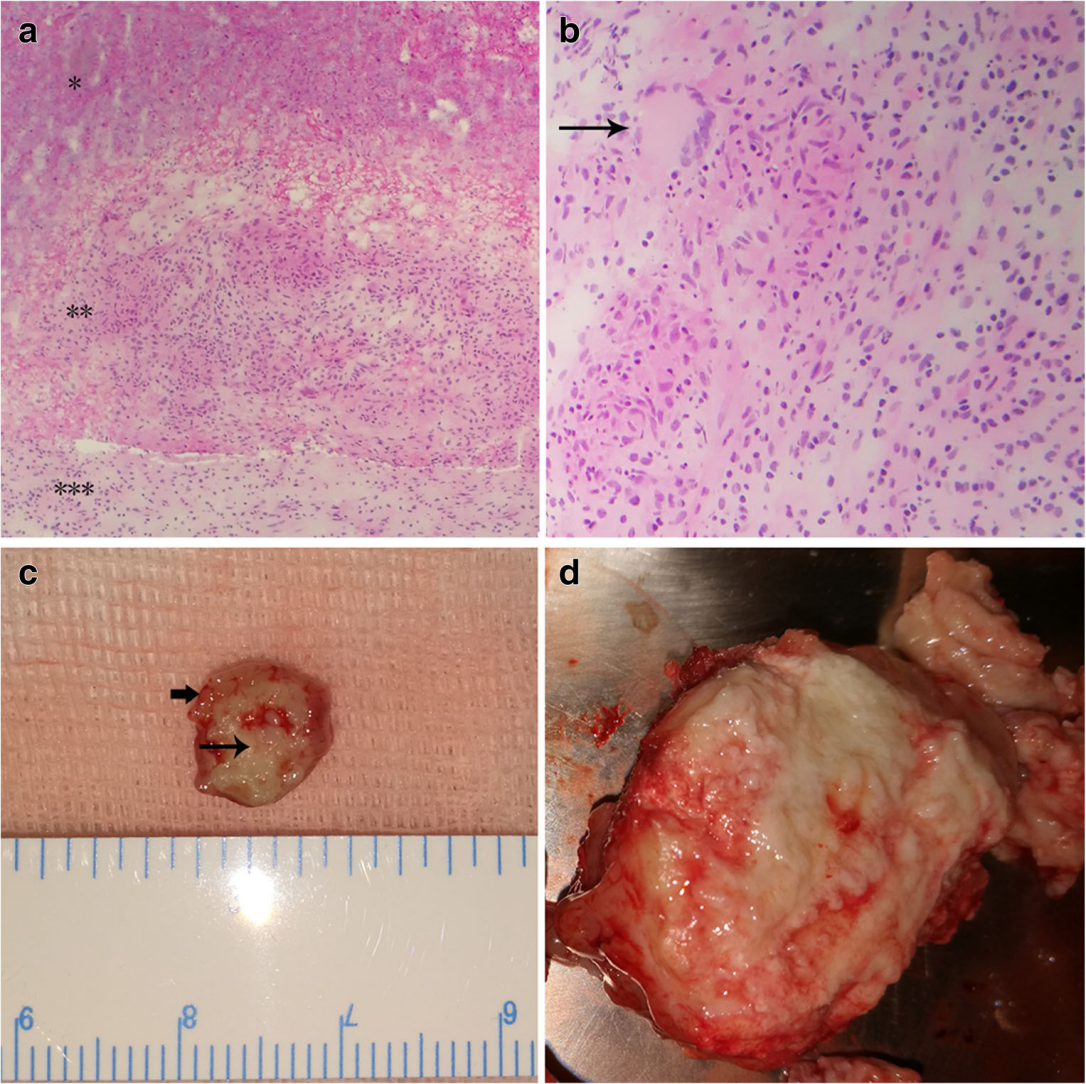
Pathological findings of tuberculoma. **a**, hematoxylin-eosin staining of tuberculoma (×100). Caseous necrosis is represented by homogenous eosin staining in the upper part *; granuloma is in the middle, represented by infiltration of neutrophils, lymphocytes and epithelia cells**; edematous neural tissue is in the bottom***. **b**, hematoxylin-eosin staining of tuberculoma showing Langhans giant cell, arrow (×200). **c**, gross specimen of tuberculoma from T12-L1 in Patient 8 showing outer layer of granuloma (short arrow) and inner part of necrosis (longer arrow). **d**, part of the tuberculoma from Patient 4 showing whitish cross section of the giant tuberculoma with limited blood supply

Cerebellar lesion was resected via paramedian suboccipital approach in five patients. Basal ganglia lesion was resected via trans-cortical frontal horn approach in two patients. Intra-spinal lesion was resected via trans-laminar approach in one patient. Specimens were sent for pathological examination, which revealed caseous necrosis and TB granuloma (Fig. 2).

Postoperative course was uneventful in seven patients except for the one who previously underwent EVD procedure. Two patients with basal ganglia mass remained stable compared with their preoperative hemiplegia. The one with intra-spinal lesion had improved lower limb strength, and the pain disappeared. Hydrocephalus was resolved in five patients.

Follow-up ranged from 10 months to 24 months. Of the eight patients, six returned to normal life after anti-TB treatment. One patient’s cerebellar lesion was resected but who’s thalamic one was preserved due to deep location and hemiparesis needed some assistance in daily life. The size of the thalamic lesion eventually decreased after prolonged anti-TB treatment.

The patient who needed EVD experienced persistent meningitis and hydrocephalus and eventually required VPS (Fig. 1). We consulted doctors from the TB ward before and after the craniotomy. Considering the lack of bacterial, serological, and PCR evidence and formal pathological report, the anti-TB treatment was not approved by the TB physician. The patient underwent VPS days after craniotomy, and then his clinical condition deteriorated. Chest CT showed lung infection, which was suspected as TB. After discussion with pediatric ICU intensivists, the lead surgeon decided to initiate anti-TB therapy. The patient’s ventricles became enlarged again after shrinkage shortly after VPS. A second EVD was performed to save the patient. Later, the CT scan showed unchanged ventricles and increased amount of pus in major cisterns. However, after four procedures under general anesthesia (EVD, cerebellar craniotomy, VPS and 2nd EVD), the patient could not survive without a ventilator, and gradually deteriorated into coma. Such deterioration was suspected to be due to TB spread and brainstem inflammation. The patient’s parents finally refused further treatment, and he died after discharge.

## Discussion

TB was once considered to be totally controlled from the 1960s to the 1970s. However, with the advent of acquired immunodeficiency syndrome (AIDS) and the increasing use of immunosuppressants, TB is back in the limelight. According to World Health Organization, TB is one of the leading causes of death globally. In 2017 alone, more than 10 million people suffered from TB, and more than 1.6 million deaths were caused by this disease. Also in 2017, about 100,000 Chinese children were affected by TB, accounting for about 11.2% of all TB patients in China [8].

TB is most fatal when it affects the CNS (CNS-TB). Without proper treatment, up to 50% of all CNS-TB patients die. Hydrocephalus and meningitis are common in CNS-TB, whereas tuberculoma is very rare, occurring in only 5% of pediatric CNS-TB patients [9]. Management of TB meningitis and hydrocephalus has been extensively studied [1, 9, 10], but the clinical feature and treatment of CNS tuberculoma is not well understood.

Six of the eight patients came from under-developed areas in the southwest part of China, where prevalence of TB is higher due to insufficient medical resources and animal husbandry. However, two patients came from local rural places, suggesting that TB prevalence exists outside urban areas and cannot be overlooked. Most tuberculomas in our cases affected the cerebellum, although the basal ganglia, thalamus, or spinal cord was not immune. One possible explanation may be the different blood perfusion rate. Blood flow in cerebral grey matter is estimated to be 80 mL/100g/min, whereas the value in cerebellar grey matter is only about 40 mL/100 g/min [11, 12]. Lower tissue perfusion leads to worse oxygenation and nutrients; thus, the patient’s immune response is not sufficiently active to prevent and clear infection [13].

The most prominent symptom involving intracranial tuberculoma is headache, which is due to either increased intracranial pressure or meningitis. Similar to the results obtained by Srikanteswara et al., the pain is dull and whole cranial in nature [14]. Vomiting is not a prevailing symptom, suggesting that intracranial hypertension progressed insidiously in most cases. Only two patients had low fever and sweating, but none of the two had prior history of lung TB or associated treatment. Of the three patients who had prior anti-TB treatment, none had fever or sweating. These findings indicated that symptoms typical of TB, i.e., low fever, weakness, or sweating, may be absent in most cases with CNS tuberculoma and that previous anti-TB treatment may further obscure these symptoms. Thus, careful evaluation of patient’s history is important in diagnosis.

MR imaging remains indispensable in the proper diagnosis of CNS tuberculoma. In our case series, most giant tuberculomas were characterized by ring enhancement pattern, low T2 intensity, and significant peripheral edema. According to Kumar et al., the pathophysiological basis for these features is as follows [15]. The ring enhancement is due to granuloma formation in the outer rim of lesion; the low T2 signal suggests caseating solid granuloma; and the peripheral edema with enhanced projections from the outer rim is possibly due to granulomatous vasculitis in the surrounding parenchyma. Our pathological section with HE staining supported this notion. Under the microscope, the adjacent neural tissue outside the granuloma was edematous, as evidenced by decreased eosin staining. The outer layer of tuberculoma was formed by the granulomatous tissue, as supported by increased infiltration of neutrophils and lymphocytes and the appearance of Langhans giant cells. The deep part of tuberculoma was characterized by homogenous eosin staining, suggesting caseous necrosis. Acid-fast staining for TB bacilli is not reliable, because in almost all tuberculoma cases, the result is negative [16]. The culture of mycobacteria from CSF is gold standard, but the positive rate (5–58%) varies and remains considerably low, and the positivity in pediatric patient is even lower (10–20%) [10, 17]. To increase positivity in smear or cultures, a minimum of 10% of CSF volume should be obtained for analysis [10]. PCR test is sensitive and can confirm *Mycobacterium tuberculosis* DNA detection in CSF, but this conclusion is based on en-plague-shaped CNS tuberculomas [17]. For giant tuberculomas, surgical resection is the treatment of choice, and a pathological study along with epidemiology, history, and MRI is sufficient to establish the diagnosis.

Approximately more than 80% of all pediatric patients with CNS TB develop hydrocephalus [18]. Treatment includes VPS, ETV, EVD, or dehydrating agents and steroids. Modified Vellore grading system is a widely validated system that can be used to predict outcome. High grades indicate poor outcome. In a systemic review conducted by Rizvi et al., around 78.6% of grade I patients, 68% of grade III patients, 65.4% of grade II patients, and only 31.5% of grade IV patients had good clinical outcome [7]. A randomized study showed that VPS was more successful than ETV in managing TB hydrocephalus (68% vs. 42%), but the complication rate was higher for VPS [19]. In our cohort, most patients presented with giant tuberculoma, and hydrocephalus was obstructive due to the mass effect in the posterior fossa or third ventricle. Except for the one treated with EVD, hydrocephalus was completely resolved for the other five patients after the resection of the lesion. MR scan of the patient who underwent EVD showed enhanced prepontine cistern, and later, CT scan showed pus collection in all major cisterns after tuberculoma resection. VPS was not helpful in this patient because of the serious spread of meningitis and lung infection, which is highly suspected as TB. Thus, most concomitant hydrocephalus cases in giant tuberculoma patients are resolved merely after lesion resection, and no additional treatment was needed. However, if TB spreads throughout or outside the CNS, then the outcome is often fatal.

There is no doubt that giant CNS tuberculoma warrants surgical resection. For multiple lesions, the resection of most space-occupying lesions is the goal of surgery. The remaining smaller lesions or those located deeply in the critical site can be managed conservatively, if the associated symptom or neurological deficit is minimal. Based on various guidelines, the recommended first-line treatment for TB meningitis consists of four drugs, namely, isoniazid, rifampicin, pyrazinamide, and ethambutol, comprising the so-called HRZE regimen [10]. For tuberculoma, medical treatment remains unvalidated. Most tuberculoma patients harbor concomitant TB meningitis; thus, similar HRZE therapy should also be applicable. A more important issue is when to initiate anti-TB treatment. Most of our patients were in a stable clinical condition during the perioperative period. They were referred to a special physician or resumed their previous therapy. However, for patients with deteriorating or emergent situation, timing of anti-TB treatment is crucial. The case of the patient who died reflects that in highly specialized tertiary medical centers, multidisciplinary therapy needs to be reinforced. Indeed, considering the toxic effect of anti-TB drugs and proper monitoring in follow-up, the prescription should be restricted to specialized physicians, who depend on concrete laboratory evidence. However, for our case, we suggested that when a neurosurgical patient is highly suspected of TB in the perioperative period, empirical anti-TB therapy should be initiated despite the lack of laboratory evidence [10]. The operation, intubation, and administration of corticosteroids during the perioperative period could contribute to the worsening of TB. Anti-TB drugs may prevent such deterioration in the patient’s condition.
